# Evaluating an Enterprise-Wide Initiative to enhance healthcare coordination for rural women Veterans using the RE-AIM framework

**DOI:** 10.3389/frhs.2023.1237701

**Published:** 2024-01-12

**Authors:** Mark R. Relyea, Rebecca L. Kinney, Eric C. DeRycke, Sally Haskell, Kristin M. Mattocks, Lori A. Bastian

**Affiliations:** ^1^ VA Connecticut Healthcare System, West Haven, CT, United States; ^2^ Department of Psychiatry, Yale School of Medicine, New Haven, CT, United States; ^3^ VA Central Western Massachusetts Healthcare System, Leeds, MA, United States; ^4^ Population and Quantitative Health Sciences, University of Massachusetts Chan Medical School, Worcester, MA, United States; ^5^ Department of Internal Medicine, Yale School of Medicine, New Haven, CT, United States

**Keywords:** women Veterans, care coordination, rural women, maternity care, preventive screening, women’s health coordination, Rural Health coordination, Veterans Affairs

## Abstract

**Introduction:**

The Veterans Health Administration (VA) Office of Rural Health (ORH) and Office of Women's Health Services (OWH) in FY21 launched a three-year Enterprise-Wide Initiative (EWI) to expand access to preventive care for rural, women Veterans. Through this program, women's health care coordinators (WHCC) were funded to coordinate mammography, cervical cancer screening and maternity care for women Veterans at selected VA facilities. We conducted a mixed-methods evaluation using the RE-AIM framework to assess the program implementation.

**Materials and methods:**

We collected quantitative data from the 14 program facilities on *reach* (i.e., Veterans served by the program), *effectiveness* (e.g., cancer screening compliance, communication), *adoption, and maintenance* of women's health care coordinators (WHCC) in FY2022. *Implementation* of the program was examined through semi-structured interviews with the facility WHCC funding initiator (e.g., the point of contact at facility who initiated the request for WHCC funding), WHCCs, and providers.

**Results:**

*Reach*. The number of women Veterans and rural women Veterans served by the WHCC program grew (by 50% and 117% respectively). The program demonstrated *effectiveness* as screening rates increased for cervical and breast cancer screening (+0.9% and +.01%, respectively). Also, maternity care coordination phone encounters with Veterans grew 36%. *Adoption*: All facilities implemented care coordinators by quarter two of FY22. *Implementation*. Qualitative findings revealed facilitators and barriers to successful program implementation and care coordination. *Maintenance*: The EWI facilitated the recruitment and retention of WHCCs at respective VA facilities over time.

**Implications:**

In rural areas, WHCCs can play a critical role in increasing *Reach* and *effectiveness.* The EWI demonstrated to be a successful care coordination model that can be feasibly *Adopted*, *Implemented*, and *Maintained* at rural VA facilities.

## Introduction

### Scope of problem

The Veterans Health Administration (VA) is the largest integrated healthcare system in the United States, serving approximately 9 million Veterans each year (1). Women Veterans comprise only 8% of the VA population yet are the largest growing demographic at VA (2). Although VA offers a range of women's health services, smaller rural VA facilities may rely on the non-VA Community Care Network (CCN) which contracts with two major third-party administrators (Optum and TriWest) to provide certain gender-specific services (e.g., mammography) to women Veterans in the community. Smaller VA facilities may not have a large enough patient population to meet the set number of screenings required to obtain certification for mammography. Additionally, all VA facilities rely on community providers enrolled in CCN for obstetric care. As women Veterans move between the two healthcare systems (VA and non-VA), coordination of care is vital to ensure that medical information is communicated in a timely and efficient manner and to help Veterans face challenges such as finding and accessing care and understanding benefits (3–6).

### Care coordination

Within the VA, designated care coordinators have been demonstrated to be a promising approach to improving access, continuity, and quality of health care among Veterans (7). Specific to women's health, studies have shown maternity care coordinators had a positive impact on helping Veterans find non-VA providers, access VA and non-VA resources, understand their benefits and coverage, assist with billing, and coordinate medical care and mental health services (8, 9). Additional qualitative work examining care coordinators for women's health more broadly has found coordinators can increase women Veterans' awareness of VA and community resources, provide education to providers and staff, and improve greater continuity of care (10). Yet, the literature has also identified challenges to coordinating women Veteran's health such as undefined roles, a lack of community provider knowledge surrounding VA services, fragmented communication between care settings, and limitations with computer systems that track care coordination (11, 12). In total, coordinators appear promising for women's Veteran's health yet successful implementation is challenging.

### Description of program

Given the prior successes of care coordination at VA, including for maternity care, the Office of Rural Health (ORH) and Office of Women's Health (OWH) launched an Enterprise-Wide Initiative (EWI) to support Women Veterans' health care coordination and management. The goal of the Women's Health Care Coordination and Management program was to expand access to care for women Veterans residing in primarily rural areas through the hiring of women Veteran Care Coordinators (WHCCs) and medical support staff. WHCCs were conceptualized as nurses that would work with providers and women Veterans to facilitate women Veteran's access to care, coordinate care, track screening and results, and reduce barriers to care. Through the grant, WHCCs could serve as mammogram coordinators, pap coordinators, maternity care coordinators, or any combination of the three. In FY21, the second round of the WHCC program was launched at 14 grantee facilities for a 3-year timeframe.

### Description of evaluation

We conducted an evaluation to support the development, and assess the impact, of the second round of the WHCC program. While prior work has examined the impact of coordinators on maternity care, the current evaluation expanded these efforts to robustly evaluate coordinators for women's health more broadly. This mixed methods evaluation used qualitative interviews and quantitative assessment to (1) Assess VA staff and provider perceptions, experiences, and expectations for the care coordinator position. (2) Evaluate the quality of gender-specific cancer screening and maternity care services with VA care coordination programs.

Our evaluation used the RE-AIM (reach, effectiveness, adoption, implementation, and maintenance) framework to structure program findings and evaluate goals. RE-AIM is a widely used evaluation framework for assessing the implementation of public health interventions and translation of evidence-based practices into the real world (13). We defined *reach* in two ways—the number of women Veterans that facilities report the program is servicing and the number of completed encounters per women Veteran served. We also examined these specifically for rural women Veterans. To assess *effectiveness*, we used three metrics: (1) Cancer (breast and cervical) screening compliance rates, (2) timeliness of communicating cancer test results (abnormal and normal), and (3) the number of contacts that maternity care coordinators had with pregnant or postpartum Veterans. We defined *adoption* of the program as the onboarding of care coordinators at program facilities. We also conducted qualitative interviews with facility WHCC points of contact that initiated funding, WHCCs, and providers to comprehend the barriers and facilitators to adoption and *implementation*. Lastly, we defined *maintenance* as the retention of the WHCC at the respective facility.

## Methods and materials

### Participants

**Figure 1:**
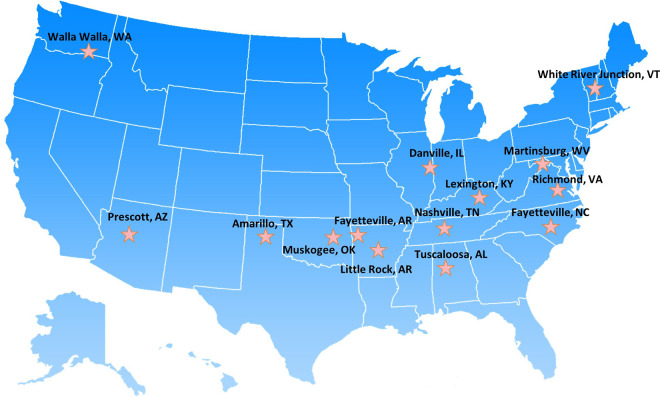
Map of 14 facilities that participated in the women Veteran care coordination program from FY21–FY22.

Funding to hire a WVCC was provided to 14 VA facilities reporting 50% or more enrolled Veterans at the facility living in rural or highly rural areas. VA uses the U.S. Department of Agriculture Rural-Urban Commuting Areas (RUCA) system to define rurality that is based on population density and linkages to urban areas (14). Geographic breakdown included the following 14 facilities: the Northeast (*N* = 1), the South (*N* = 8), the Midwest (*N* = 4), and the West (*N* = 1; see Figure 1). For our comparison group, we examined nationwide VA data from 130 facilities. This evaluation was a quality improvement project designed to support VA operations and thus was not research.

### Measures

#### Reach

We defined reach as (1) the number of women Veterans (and rural Veterans) served by the program, defined by ORH as the number of unique Veterans directly touched or impacted by the ORH funded program and (2) the number of encounters per women Veterans (and rural Veterans) served. Encounters were defined by ORH as the count of (a) phone calls between coordinators and patient, (b) face to face encounters between coordinators and patient, (c) mammograms or paps scheduled, (d) women Veterans being tracked or followed or any combination of the above items. Facility contacts provided this information quarterly through online VA systems (i.e., the Office of Rural Health Management & Analysis Tool [OMAT] and New ORH Management and Analysis Database [NOMAD]). We examined the sum of all women Veterans reached across facilities during the first two years (FY21, FY22) of the EWI. We chose to examine reach in half year increments beginning in Q2 because facilities had a lag in the hiring and onboarding of WHCCs and to avoid minor quarterly fluctuations.

#### Effectiveness

We defined effectiveness as the percentage of eligible women Veterans that were provided breast cancer screening and cervical cancer screening, timeliness of communicating cancer test results, and the number or contacts maternity care coordinators had with pregnant and post-partum Veterans. We used electronic health record data provided through the VA VSSC dashboard reports to determine both breast cancer screening compliance and cervical cancer screening compliance for all 14 facilities between FY 2020 (prior to funding) and FY 2022. For context, we also provide national VA rates. Timeliness was acquired through the Communication of Test Results (CTR) reports which examined if (a) results are communicated to patient and documented in EHR and (b) if patient received results in a timely fashion according to guidelines (i.e., 30 days for normal results and 7 days for abnormal results). We received CTR reports from FY22 Q1 and Q2 only. The number of contacts that maternity care coordinators had with pregnant and postpartum women was provided by facility contacts quarterly in online systems (i.e., OMAT, NOMAD).

#### Adoption

We defined adoption as the onboarding of WHCCs. We also described the variation in the role of coordinators across the domains of mammography, reproductive health (cervical screening), and maternity care maternity care. Facility contacts reported data quarterly in online systems (i.e., OMAT, NOMAD) and vacancy reports.

#### Implementation

To assess implementation barriers and facilitators, we conducted semi-structured interviews with the 14 facility WHCC points of contact that initially requested funding (12 were women Veteran program managers, one was a medical director, and one was a women's health nurse navigator) two were heads of the women's health clinic), WHCCs, and providers. We asked WHCCs to describe care coordination for preventive screenings and maternity care across four phases: tracking, scheduling, obtaining results, and follow-up. Additionally, we asked coordinators their perceptions of facilitators and barriers to coordinating women veterans' care between the VA and non-VA settings. One member of the research team conducted all interviews by Teams. Our qualitative methods have been previously outlined and published elsewhere, but in short, each interview was digitally recorded and transcribed verbatim (10).

#### Maintenance

We defined maintenance by the retention of care coordinators following onboarding. Facility contacts reported data quarterly in online systems (i.e., NOMAD) and vacancies reports. Additionally, we supplemented these reports with the qualitative information collected during the interviews.

### Data analysis

We conducted descriptive statistics to examine the program reach and the frequency of encounters per woman Veteran. To examine effectiveness, we conducted descriptive statistics to examine change over time in rates of screening, timeliness of communicating test results and contacts with pregnant and post-partum women. We also provided each of the rates at VA overall for context. However, we did not make direct comparisons to the VA overall as the primary goals were change within facilities, facilities were self-selected, and we did not have matched comparison sites. Qualitative transcripts were independently coded, summarized, and achieved consensus among two qualitative coders pertaining to the underlying themes for the final dissemination (10).

## Results

### Reach

**Figure 2:**
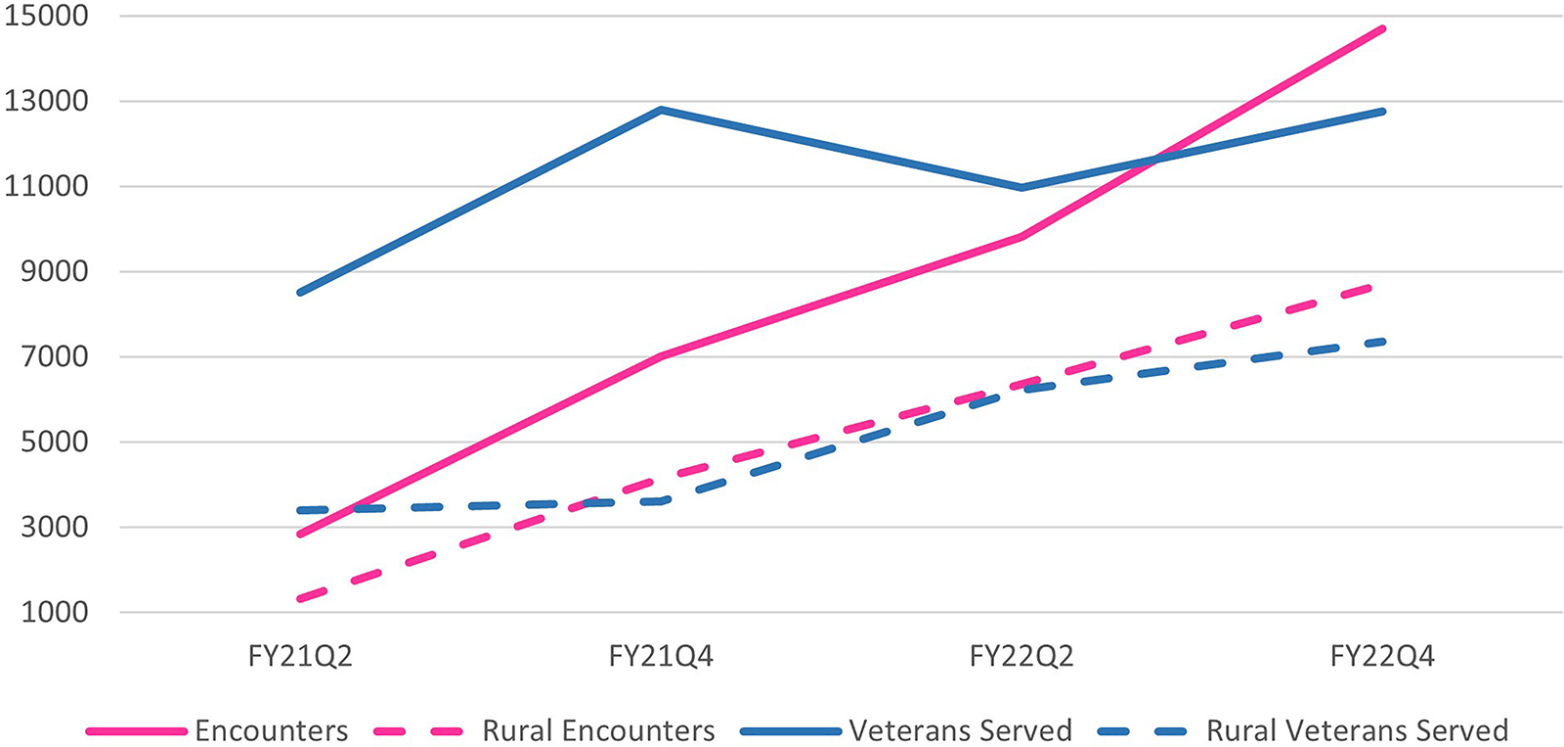
Reach of women Veteran care coordination program at 14 VA facilities from FY21–FY22. Encounters = the number of face-to-face patient encounters, patient phone calls, mammograms or paps scheduled, patients being tracked or followed, or any combination of those items. Served = the number of unique Veterans directly touched or impacted by the ORH funded program. Numbers reflect half year totals at each timepoint; FY21Q2 = from October 1, 2020 through March 31, 2021, FY21Q4 = April 1, 2021 through September 30, 2021, FY22Q2 = numbers from October 1, 2021 through March 31, 2022, FY22Q4 = April 1, 2022/through September 30, 2022.

In FY21 Q2, grantee facilities reported serving 8,520 women Veterans (range 19 to 2,840 at each site) with 39.7% of women Veterans living in rural areas. Nine sites did not report data FY21 Q2 as they had not yet onboarded coordinators. By end of second year of grant FY22Q4, facilities reported serving 12,761 Women Veterans (range 60–3,609 at each site) with 57.6% of women Veterans living in rural areas (Figure 2). Similarly, encounters increased from 2,838 in FY21Q2 (range 8–1,607) to 14,701 in FY22Q4 (range 28–4,763) with rural women Veterans accounting for 46.4% in FY21Q2 and 59.2% in FY22Q4. At the beginning of program, facilities reported 0.3 encounters per women Veteran and ended with 1.2 encounters per women Veteran; likewise, rural encounters reported as 0.4 per Veteran and 1.2 respectively). Women Veterans served increased by 50% during this grant time and encounters increased over 400% with rural Veterans seeing larger increases of over 100% and over 550%.

### Effectiveness

**Figure 3:**
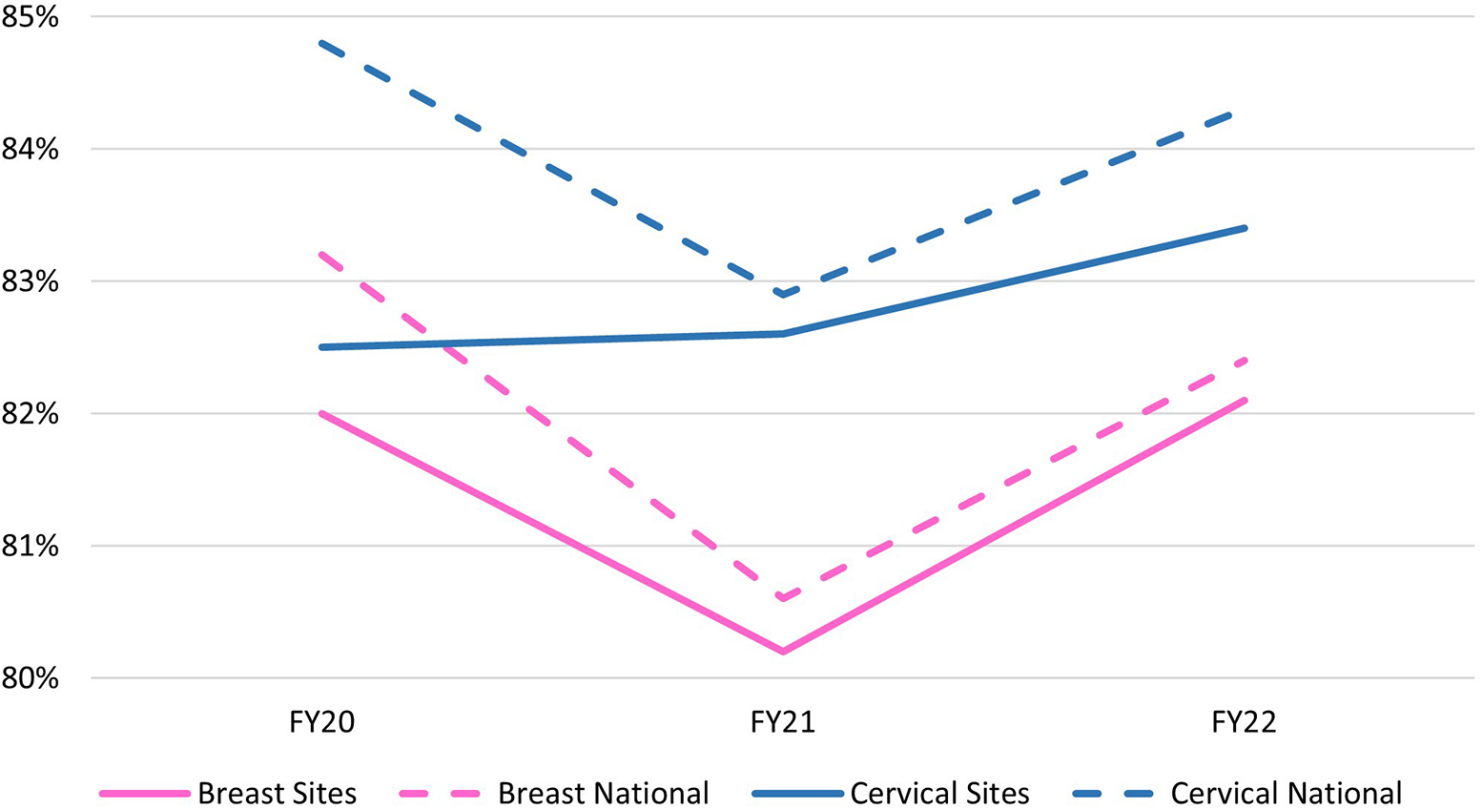
Breast and cervical cancer screening rates FY20–22 at 14 VA grantee facilities vs. national VA comparison.

The VSSC data showed over 80% screening rates for both breast and cervical cancer at the 14 grantee facilities. Breast cancer screening rates at the 14 facilities remained steady between FY20 and FY22, from 82.0% to 82.1%, with a small drop in FY21 (Figure 3). For context, the VA national average showed a decrease during this period from 83.2% to 82.4%. Cervical screening rates at grantee facilities also increased, from 82.5% in FY20 to 83.4% in FY22. National VA rates slightly decreased from 84.8% to 84.3% during the same time frame.

The grantee facilities communicated 100% of mammogram test results, including both normal and abnormal results. For context, nationally VA communicated 99.3% of normal results and 94.4% of abnormal test results. Regarding cervical screening tests, grantee facilities reported 97.2% of normal results and 98.2% of abnormal results. VA national rates were 98.2% for normal results 91.5% for abnormal cervical test results. For both tests, notification of abnormal results (∼92% to 95%) was lower than notification of normal results (∼98% to 100%) due to shorter notification time frame.

In FY22, facilities began reporting number of maternity calls per quarter. In Q1, there were 1,312 calls by the end of Q4 the calls increased to 1,785. In one calendar year, calls increased by 36% as maternity care continues to be a priority and area of growth for VA.

### Adoption

By the second quarter of fiscal year (FY) 2022, 100% of the funded facilities had WHCCs hired and operational. Facilities varied in the types of coordinators adopted in order to fit local needs. Out of the 14 facilities, eight (57.1%) used the program funds to hire a coordinator to focus on mammography, reproductive health (cervical screening), and maternity care, two (14.2%) hired a coordinator specific to maternity care and reproductive health (not mammography), one (7.1%) hired a mammography and reproductive health coordinator (not maternity care), and one (7.1%) hired a maternity care coordinator only. Thirteen (92.8%) of the coordinators hired were Registered Nurses (RNs) and one was a Licensed Practical Nurse (LPN). Additionally, two facilities also used fund to hire a medical support assistant. Qualitative data indicated that the hiring process at majority of facilities was impeded by the pandemic, but once restrictions were lifted the WHCC positions were filled rather rapidly. The pandemic presented a second challenge to adoption, as the retention of the new hires in their WHCC roles was often challenged by the competing priorities resulting from the public health emergency. One facility reported their WHCC was detailed to the emergency department in Q1 of FY 2022 to assist with COVID. So, it is plausible that WHCC staffing challenges may return if COVID resurges.

### Implementation

Program implementation was driven by three underlying factors; (1). the facilitators of, (2). the barriers to, and (3). the impact of the COVID-19 pandemic on women health care coordination. At varying timepoints, the qualitative team conducted stakeholder interviews with various VA clinical staff across the facilities to assess program implementation fidelity, facilitators, and barriers. It was reported that prior to WHCC funding, women's health care coordination was piecemeal, and Veterans were often falling through the cracks. Hence, early on in the implementation stage, the role of the WHCC was defined by the need at each respective facility. For example, facilities that already had someone coordinating the cancer screenings, opted to have the WHCC focused on maternity care coordination. Alternatively, facilities serving a larger women Veteran population implemented a team approach in which cancer screening and maternity coordination was to be divided between two WHCCs and staff (e.g., MSA, RN, etc.). Stakeholders reported that a comprehension of the resources available and the gaps in women Veterans' health care coordination guided implementation and led to more successful WHCC models at each facility.

#### Facilitators of WHCC implementation

Facilitators of WHCC program implementation included a collaborative team approach, strong relationships with the site's Office of Care in the Community (OCC, or the local VA office that aids eligible Veterans in receiving health care from providers outside of a VA medical facility), institutional and facility support, and training in VA's System for Mammography Results Tracking (SMART). Qualitative interviews revealed a collaborative approach to coordination that was unique to the funded facilities. Facilities were thoughtful in developing a team(s) or model(s), rather than just one position, to address women's' health care coordination. Facility-specific care coordination models often incorporate one or more of the following: a nurse navigator, pharmacist, social worker, a medical (MSA)/program (PSA) support assistant, and/or a community care representative. One provider underscored the nature of her collaboration with the WHCC, “*I can’t function normally and peacefully without [coordinator] at my side, honestly*”.

When the WHCC had an established line of communication with the Office of Care in the Community (OCC), they were more likely to report successful coordination efforts at all stages of women Veterans' preventive health care delivery. One WHCC underscored the utility of her relationship with the OCC, “[we are] increasing communication so we are proactive not reactive…” A few facilities did not have established relationships with OCC noting that this unmet facility need.

Strong institutional and facility level support influenced the success of the WHCC implementation. Conversely, WHCCs who reported less facility level support described being pulled away from their coordination efforts at times to serve other priorities, “I am often asked to take on other primary care priorities leaving me little bandwidth for my coordination responsibilities”. The coordinators at two facilities claimed that “for a long-time women's health was on the back burner… resulting in some barriers to efficiently coordinating care…I now more closely see the administrative side…women's health was not supported well”. WHCCs voiced that greater leadership support was essential to successful program implementation.

#### Barriers to WHCC implementation

Barriers to WHCC implementation included inadequate space and staff, having mammography off site, and untimeliness in getting results back from non-VA providers. The allocation of space and staff were the two most-commonly reported unmet needs to delivering women Veterans' health care at VA facilities. WHCCs reported needing more clinic space as the reach to women Veterans increased, “it is really hard to do a pap smear given the rooms are so small and uncomfortable”. Provider recruitment and retention was a common barrier to coordinating care, “we are lacking a female OB/GYN, many Veterans will leave our facility because they prefer a women provider”. Providers confirmed that as their facilities engaged more women Veterans in VA care, there was a dire need from more space and staff to facilitate this growth.

While communication served as a facilitator to WHCC implementation, it could also be a barrier when it was lacking. WHCCs reported that when they did not have fluent communication with the VA OCC and/or community providers, they experienced barriers to finding a provider for the Veteran and/or get screening results back in a timely manner. A few coordinators were still trying to improve the process, “on average it takes a couple of weeks to get results back…at the very least, it would be helpful if both the VA and OCC providers were on the same software”.

The providers were quick to point out how integral the WHCC implementation has been to the continuity of women Veterans' care, specifically, how the new position(s) facilitated more communication between the VA, OCC, and the community providers. At most facilities, the WHCC helped identify community providers and ensured that proper patient reports and information were exchanged, which relieved clinicians of this burden and stress.

#### Impact of COVID on implementation

The unforeseen circumstances surrounding the pandemic resulted in barriers to WHCC implementation at both screening compliance and staffing levels. During the pandemic, women Veterans' cancer screenings were put on hold for a period of time. Simultaneously, many WHCCs, providers, and staff were detailed to other clinical priorities further hindering program implementation. By April 2021, most facilities were starting to catch-up on screening numbers as patients were slowly being scheduled to come back for appointments. WHCCs reported sending out reminder letters and making calls to encourage Veterans to come in for preventive screenings to keep numbers compliant. Many providers reported that the WHCC was pivotal to re-engaging women Veterans in their preventive care, post-pandemic. One provider commented,

“*…women were falling in the cracks [for mammography screening]and it was a big problem, but since we have the new coordinator, we are catching up and bringing everything up to date and I can't even imagine [coordinator] not being here and helping me out*”.

Similarly, as women Veterans relied on community obstetricians for maternity care, yet often continued to receive VA care for other conditions, such as mental care health, during their pregnancies, WHCCs who focused on maternity care were essential to coordinating the VA and community care that pregnant Veterans needed in a timely manner within the restraints of the pandemic. In the capacity of maternity care coordination champion, the WHCCs aided women Veterans in addressing some of the unique challenges of simultaneously using two health care delivery systems, including appointment scheduling, sharing of results, and finding a provider in the network.

### Maintenance

Once all facilities had adopted the WHCC positions, the positions were maintained through the end of the second year. EWI WHCC program maintenance was influenced by leadership support at both the facility and institutional levels. Coordinators may have additionally impacted the maintenance of other providers as the majority of providers interviewed reported higher satisfaction, less burnout, and more time for patient encounters as they shifted some their administrative tasks to the WHCCs.

## Discussion

The ORH/OWH Enterprise Wide Initiative program's aim was to increase access to and continuity of preventive care to women Veterans through the utilization of VA women's health care coordinators (WHCC) in rural areas. Using the RE-AIM framework as a guide to our comprehensive evaluation, the WHCC program appeared an effective model which increased women Veterans' access to and continuity of care within the VHA. In the subsequent paragraphs, we discuss the implications of women Veterans health care coordination and underscore some considerations for future coordinator assessments that seek to apply the RE-AIM framework.

Our findings support those of prior work that have demonstrated the potential benefits of women Veterans' health care coordination (9). In line with our prior work focused on women's health care coordinators (10), we found that the WHCC program expanded reach. The most substantial gains in Veteran reach came after a reduction in the impact of the pandemic making it difficult to tease out whether results were program-specific (e.g., due to funded WHCCs enabling greater capacity for screening and outreach) or a result of the loosening of pandemic restrictions (e.g., an increase in scheduled screenings that were reduced during the pandemic). Yet, cervical and breast cancer screening compliance increased at the grantee facilities during a time in which the VA nationally exhibited decreases. These findings may be reflective of the WHCCs' impact on access and timeliness to care among rural women Veterans. Given that Veterans in rural areas face additional barriers to care (5), the relative increases compared to the VA average indicate these improvements would have been less likely without the additional support of the WHCCs. The finding that Veterans initially had less than one encounter on average was unexpected yet may have been due to ORH/WHC Enterprise Wide Initiative definitions for the number of Veterans “served” and “encounters” that were broad enough to allow WHCCs to capture the diverse ways they interacted with Veterans (e.g., “encounters” included any combination of direct patient care, scheduling, and tracking). This flexibility may have enabled WHCCs to report that they were serving more Veterans than had encounters if they applied a broader definition for served (e.g., including Veterans being tracked) than they did for encounters (e.g., face to face encounters or screenings).

The RE-AIM framework also highlighted broader lessons to be considered when implementing the WHCC positions at future VA facilities. The first lesson emphasized the of importance examining *Reach* through both quantitative and qualitative data. Given that women Veterans are the numerical minority in the VHA, their healthcare needs often are referred out to providers in the community or go unmet. Defining the program's *Reach* was an initial challenge due both to difficulty determining the number of women Veterans, especially rural Veterans, who were eligible for VA care in many of the rural locations and due to the variety of ways coordinators may interact with Veterans (e.g., through direct encounters, tracking, and scheduling) Iterative feedback, which is an essential component of the RE-AIM framework, guided the early stages of our evaluation in developing a pragmatic approach to understanding reach. Through discussions with ORH and OWH as well as lessons learned through our prior evaluation, we concluded that reach was best defined by utilizing self-reported coordinator estimates based on definitions for Veterans “served” and “encountered” that were flexible enough to cover the diverse roles of coordinators and through enabling WHCCs to report how many of these Veterans were rural (10). The current evaluation used VA's NOMAD system to quantitatively track facilities' self-reported reach to rural women veterans, as well as the number of completed encounters per veteran served. This method of determining reach was efficient and appeared sensitive to change. Grantee facilities demonstrated an increase in reach to all women Veterans served and the number of rural and highly rural Veterans served.

While our quantitative findings demonstrated an increase reach at the facility level, provider interviews confirmed these results with firsthand accounts of how the coordinators had expanded reach. Providers were quick to report that coordinators had led to an increase in new and returning women Veterans at their facilities. Furthermore, by examining the increase in the representation of rural women Veterans served by the program out of all Veterans served by the program, the evaluation team was able to examine the impact of the WHCC program on patients who may have otherwise “fallen through the cracks”.

The RE-AIM model enabled evaluating *effectiveness* at the Veteran, provider, and facility levels. The pandemic presented a challenge during the first quarter of WHCC program implementation, as strict protocols impacted adherence to preventive cancer screening guidelines and in-person visits. Still, the numbers demonstrated that adherence rates at the program facilities slightly increased during a time when numbers were declining throughout the VA, nationally. Qualitative interviews further revealed the impact of the WHCC position on facilities' screening rate adherence, post-pandemic. Our evaluation team also found that the facility adherence was often indicative of WHCC's ongoing communication with patients regarding the importance of the screenings, while educating Veterans on the VA safety precautions surrounding in-person encounters, post-pandemic. Effectiveness of coordinator on facilitating care with non-VA providers was also demonstrated in the timeliness and efficiency in which screening results were reported back to the VA from outside providers. Subsequent to hiring the WHCCs, VA providers noted an increase in the uptake and timeliness of receiving mammography results back from community providers. Much of this success resulted from having a designated WHCC for following the women Veteran's community care and ensuring the any associated results/reports were received at the VA. WHCCs who developed strong collaborations and communication with the clinical teams, the local Office of Care in the Community, and/or community providers demonstrated the most positive outcomes. The WHCC program efficacy was demonstrated through a comprehensive qualitative examination of the expectations and impact of the WHCC positions at the Veteran, clinic, and facility leadership levels. The finding that WHCCs may have reduced the administrative burden of providers points to a critical potential impact that coordinators may play in increasing time for providers to focus on other tasks and possibly reduce provider burnout. Thus, although efficacy or effectiveness are often judged quantitatively, as we did here, understanding the context and facilitators of effectiveness requires mixed methods evaluation. As coordinators interact with patients, providers, and systems, the qualitative data is critical to identify the impacts of the coordinators that are unexpected or had not been identified in the initials stages of the program evaluation as primary outcomes (e.g., providers having more time for patient encounters). Additionally, qualitative information is critical to understand processes and activities of coordination that led to quantitative improvements. Lastly, qualitative information was critical to interpret the extent to which the coordinators improved outcomes above and beyond the increases seen following the lessening of pandemic restrictions. Therefore, mixed methods examination of reach and impact may be especially beneficial in evaluations of healthcare systems to interpret the signal or impact of programs from the noise of the numerous historical effects and changes that occur in large healthcare settings over time.

*Adoption* of the WHCC program model came with some challenges. The most striking lesson was the time in which it took to hire and fill the new positions, as it was not until the end of the first year that 100% of facilities reported having a WHCC in place. Once the facilities had the WHCC in place, the majority of clinical teams were quick to adapt to change. Facilities were thoughtful in developing teams or models, rather than just viewing the WHCC as one position to address care coordination. Those facilities that utilized the team approach to coordinator adoption reported fewer barriers to implementation when compared to facilities that relied on coordinator as an independent resource. The broader lesson that resulted from our evaluation was that thoughtful adaptations to program *adoption* can impact implementation and effectiveness.

Among facilities, the *implementation* plans varied and were often driven by the resources that were already in place. Collectively, the facilities provided a wealth of lessons learned in the program implementation phase. Facilities that had a clear definition of the role and responsibilities of the WHCC on the clinical team reported ease and success in the implementation. At these facilities, providers and staff recognized the value of utilizing the WHCC appropriately, as opposed to facilities in which the WHCC's utility was unclear resulting in barriers to efficiently incorporating the WHCC into the clinical team. Communication was a second attribute that led to successful program implementation at facilities. Not surprisingly, the more the WHCC strengthened communication with providers, staff, community providers, and leadership, the more successful the implementation. Our team also identified some barriers to implementation, the most dominant being lack of leadership support and limited physical space for women Veterans' health care teams. A final takeaway lesson from the implementation domain was the value of the monthly WHCC meetings held at the national level. These meetings provided a forum in which WHCCs could address the barriers and facilitators to implementation, which in return, help to inform the process at all facilities.

Similar to *Reach*, there were lessons learned over time by our team on how to comprehensively evaluate program *maintenance*. Our previous work in the realm of rural WHCCs had revealed some ambiguity surrounding the best way to assess sustainability of the program and methodically assess program maintenance (10). Subsequently, the team revised our qualitative interview guides to comprehensively query on program sustainability at earlier timepoints throughout the program implementation. An examination of program maintenance also aided in better understanding of coordinator attrition and burnout, so that these barriers could be addressed systematically in future program implementation plans.

### Limitations

The results of our study should be considered in the context of limitations. Our findings were primarily descriptive as we lacked a true control group. Although we report VA national averages for context, we did not have matched facilities for comparison and were only able to discuss changes within grantee facilities. As mentioned above, it was difficult to disentangle program effects from the impacts of the lessening of COVID restrictions. In addition to a continued use of mixed methods to understand program impacts in complex healthcare settings given historical effects, future studies could consider a stepped-wedge design allowing for a more robust test of the impact of coordinators. Data on the number of Veterans served, the number of encounters, and the number of maternity care contacts made were self-reported by facilities. In addition to the typical issues with self-report data, facilities had to track these numbers. Although reach and encounters were defined for facilities, there may have been variation between facilities in how these terms were interpreted and reported. Future research should use electronic health records to acquire or verify these data. Additionally, as we did collect patient centered feedback within the context of this evaluation, future studies seek to further understand women Veteran's perspectives on coordinated women's health services through the VA.

## Conclusion

Women's health care coordinators (WHCC) appeared to be effective in expanding access and timeliness of care to women Veterans in rural areas, particularly when the WHCCs are part of a larger collaborative clinical team. The application of RE-AIM framework facilitated a systematic method in which to collect, analyze, and apply both qualitative and quantitative data in the assessment of program reach, effectiveness, adoption, implementation, and maintenance of the WHCC program. The lessons learned from utilizing the RE-AIM approach may aid in the success of the broader implementation of the WHCC model across rural VA facilities throughout the nation.

## Data Availability

The datasets presented in this article are not readily available because the data will be shared by request and with a data sharing agreement following all regulations governing federal data. Requests to access the datasets should be directed to mark.relyea@va.gov.
